# Randomised controlled trial of a web‐based mental health, self‐harm and suicide gatekeeper resource for parents and caregivers

**DOI:** 10.1111/jcpp.70189

**Published:** 2026-06-12

**Authors:** Alison L. Calear, Sonia M. McCallum, Dominique Kazan, Michelle Torok, Aliza Werner‐Seidler, Bridianne O'Dea, Alyssa Morse, Louise M. Farrer, Fiona Shand, Philip J. Batterham

**Affiliations:** ^1^ Centre for Mental Health Research The Australian National University Canberra ACT Australia; ^2^ Black Dog Institute University of New South Wales Sydney NSW Australia; ^3^ Flinders University Institute for Mental Health and Wellbeing Flinders University Adelaide SA Australia

**Keywords:** Youth mental health, suicide prevention, help‐seeking, gatekeeper training, parents and caregivers

## Abstract

**Background:**

Parents and caregivers play key roles in recognising early signs of mental distress and facilitating access to support, yet often lack the confidence and knowledge to respond effectively. Gatekeeper training programs can improve skills, but few are specifically designed for parents. The aim of the current study was to assess the effectiveness of the *Recognise, Respond and Support – A Parent's Guide to Youth Mental Health* resource in improving parental capacity to support their child's mental health.

**Methods:**

A two‐arm randomised controlled trial was conducted with Australian parents and caregivers of children aged 5–17 years. Outcomes were assessed at baseline, post‐intervention and 12‐week follow‐up. The primary endpoint was parental self‐efficacy to recognise, respond to, and access support for mental health problems and suicide risk in their child/ren at post‐intervention. Secondary outcomes included perceived gatekeeper knowledge, mental health and suicide literacy, stigma, and help‐seeking attitudes, intentions and barriers. The trial was prospectively registered with the Australian and New Zealand Clinical Trials Registry (ACTRN12623000933651).

**Results:**

A total of 509 participants were recruited (97% female; mean age 43 years). At post‐intervention, mixed‐model repeated‐measures ANOVA indicated significant intervention effects for self‐efficacy for mental health (*p* = .04) and suicide (*p* = .01), perceived knowledge (*p* < .001), barriers to help‐seeking (*p* = .02) and suicide stigma (*p* = .02) and literacy (*p* = .01), with some effects maintained at 12‐week follow‐up. No significant changes were observed for mental health literacy and stigma, or help‐seeking attitudes or intentions.

**Conclusions:**

Gatekeeper training targeted to parents and caregivers can be effective in improving participant confidence and knowledge to support their child's mental health. Future research would benefit from identifying strategies to improve participant engagement with web‐based interventions.

## Introduction

Mental disorders, suicide and self‐harm are significant public health problems in children and adolescents. The global prevalence of anxiety and depressive disorders in children and adolescents has been reported to be 6.5% and 2.6%, respectively (Polanczyk, Salum, Sugaya, Caye, & Rohde, 2015), while the proportion of adolescents worldwide reporting suicidal thoughts or a suicide attempt in the past 12 months has been estimated to be 15.1% and 5.2%, respectively (Van Meter, Knowles, & Mintz, 2023). Given the high prevalence of mental disorders and suicide, timely identification is critical, as earlier access to treatment and support is associated with improved outcomes (Cosgrave et al., 2007).

For many children and adolescents, their parents or primary caregivers are the main source of support in times of distress, with parents often recognising changes in their child's behaviour that may indicate a problem and initiating help‐seeking to access mental health care and support (Platell, Cook, & Martin, 2023; Reardon et al., 2017; Villatoro, DuPont‐Reyes, Phelan, Painter, & Link, 2018). As potential ‘gatekeepers’ to care, it is crucial that parents and caregivers have the necessary knowledge and awareness of mental disorders and suicide risk in children and adolescents to enable them to access appropriate care and support when it is needed.

Gatekeeper training interventions are designed to improve the recognition and referral of people at risk of psychological distress or suicide. These interventions often consist of psychoeducation to increase knowledge of the signs and symptoms of mental disorders and suicide risk, skills training to increase confidence in initiating and providing immediate support and the promotion of attitudes to reduce reluctance to intervene (Burnette, Ramchand, & Ayer, 2015). Gatekeeper interventions have been found to improve confidence in supporting a person in distress, reduce stigma and increase the recognition and referral of individuals at risk of suicide or distress (Torok, Calear, Smart, Nicolopoulos, & Wong, 2019; Yonemoto, Kawashima, Endo, & Yamada, 2019). To date, few self‐directed gatekeeper training interventions have been purpose‐designed for, or evaluated with, parents or caregivers (Murphy, Heary, Hennessy, O'Reilly, & Hennessy, 2022; Torok et al., 2019). An uncontrolled trial of a generic online suicide prevention training program in a sample of parents of young people aged 12–25 years (McKay et al., 2022) found the program to be associated with increased parental self‐efficacy and formal help‐seeking intentions, as well as increased suicide literacy at 3‐month follow‐up (McKay et al., 2022). While the results of this study are positive, the constrained focus on suicide, the use of a program not explicitly tailored for parents and the uncontrolled trial design are inherent limitations.

To address these limitations, a new purpose‐designed mental health and suicide gatekeeper resource was iteratively developed in collaboration with parents and caregivers over three phases. An initial scoping survey (*n* = 631) ascertained information needs and preferences for a gatekeeper resource (e.g., modality, delivery method, target group), finding a preference for an online self‐directed resource targeted at parents of children and adolescents. Semi‐structured interviews (*n* = 14) were also conducted with parents and caregivers with a lived experience of seeking help for youth mental health concerns to further guide resource content. Lastly, prototype review and testing were conducted with parents (*n* = 9) to refine content, appearance and usability (McCallum et al., in press).

The resulting resource, called *Recognise, Respond and Support – A Parent's Guide to Youth Mental Health*, is a self‐directed and interactive web‐based resource targeted to parents and caregivers of young people aged 5–17 years. The online self‐directed format has the advantage of being scalable, flexible and accessible, enabling parents to access support at a convenient time and place while reducing barriers such as travel, childcare and stigma (Opie et al., 2024). The resource provides evidence‐based information, lived experience perspectives and help‐seeking guidance, and resources to assist parents and caregivers to support their child's mental health. Due to the online self‐directed format, the resource doesn't provide advice on individual concerns or issues. The resource takes between 90 and 120 min to complete and can be completed in one or multiple sittings. To ensure the resource was relevant to parents and caregivers both with and without children currently at risk, it was designed to have a dual focus on building knowledge and supportive attitudes prior to difficulties arising in their child, as well as teaching skills to respond and support a young person with existing mental disorders or suicide risk.

The resource consists of three key components: ‘Recognise’, ‘Respond’ and ‘Support’. The ‘Recognise’ section aims to provide parents and caregivers with the knowledge to recognise mental health problems or suicide risk in their child and assist parents in identifying when external professional help may be needed. Age‐specific information on signs and symptoms for children and adolescents is provided. The second component, ‘Respond’, is designed to provide parents and caregivers with guidance on what to do if they have concerns about their child's mental health and how to respond to a disclosure of suicidal thoughts or psychological distress, including safety planning. The ‘Support’ component of the resource aims to provide parents with information and step‐by‐step guidance on accessing professional help for their child and what to do in a crisis situation. Downloadable information sheets are available throughout the resource and direct links to these are provided at its conclusion. Further details on the resource used within this trial are available in the protocol publication (Calear et al., 2024) and resource development publication (McCallum et al., in press).

The primary aim of the current trial was to assess the effect of the Recognise, Respond and Support – A Parent's Guide to Youth Mental Health resource on participant self‐efficacy to recognise, respond and access support for mental health problems and suicide risk in their child at post‐intervention (primary endpoint) and at 12‐week follow‐up, compared to participants in a wait‐list control condition. It was hypothesised that participants in the intervention condition would report higher levels of self‐efficacy at post‐intervention and follow‐up than participants in the wait‐list control condition.

Secondary aims of the trial included (a) evaluating differences at post‐intervention and 12‐week follow‐up between the intervention and wait‐list control conditions on perceived gatekeeper knowledge, mental health and suicide literacy, mental health and suicide stigma, and help‐seeking barriers, attitudes and intentions, and (b) assessing user satisfaction and acceptability of the resource. It was hypothesised that at post‐intervention and 12‐week follow‐up, participants in the intervention condition would report higher levels of perceived gatekeeper knowledge, mental health and suicide literacy, help‐seeking attitudes and intentions, and lower levels of mental health and suicide stigma and help‐seeking barriers than participants in the wait‐list control condition.

## Methods

### Trial registration

The trial was prospectively registered (ACTRN12623000933651) with the Australian and New Zealand Clinical Trials Registry (https://www.anzctr.org.au) on 29/08/2023.

### Design

A two‐arm randomised controlled trial was conducted with outcomes assessed at pre‐intervention, post‐intervention (primary endpoint) and 12‐week follow‐up (secondary endpoint). Ethical approval was obtained from the Australian National University's Human Research Ethics Committee (Protocol 2023/195).

### Sample size, randomisation and blinding

Calculations of required sample size were based on detecting a moderate post‐intervention effect size of 0.4 for change in self‐efficacy between the intervention and wait‐list control condition, assuming a correlation of 0.5 between pre‐intervention and post‐intervention scores. Allowing for a 30–50% attrition rate and setting power at 0.90, the target sample size was 380 participants (190 per condition).

Participants were randomised to the intervention or wait‐list control condition upon completion of the baseline survey. Stratified randomisation was conducted by an independent statistician, who was not involved in the day‐to‐day management of the trial according to ICH guidelines (Lewis, 1999), using a computerised random number generator with 1:1 allocation to the intervention and wait‐list control conditions using predetermined block sizes of 6. Randomisation was stratified by participant gender (female vs. male, non‐binary, use different term, prefer not to answer) and child with experience of a mental health problem (yes vs. no) to ensure an even balance of mothers and other parents and pre‐existing child mental health exposure across conditions. The identity of participants was concealed from the statistician during this process. After randomisation, all participants were informed of their trial condition via the email address they provided in the pre‐intervention survey.

### Participants

Eligible participants were individuals aged 18 or older who were a parent or caregiver of a child or children aged 5–17 years, had access to the Internet and were located in Australia. The current mental health status of the children was not an eligibility criterion. Participants were recruited from September 2023 to September 2024 through social media platforms using paid advertisements and promotion on established Facebook groups (parenting, institutional), advertisements in school newsletters and via an established research register of parents interested in mental health research managed by the research team. Figure 1 presents the CONSORT flow diagram of recruitment and retention of participants in the trial.

**Figure 1:**
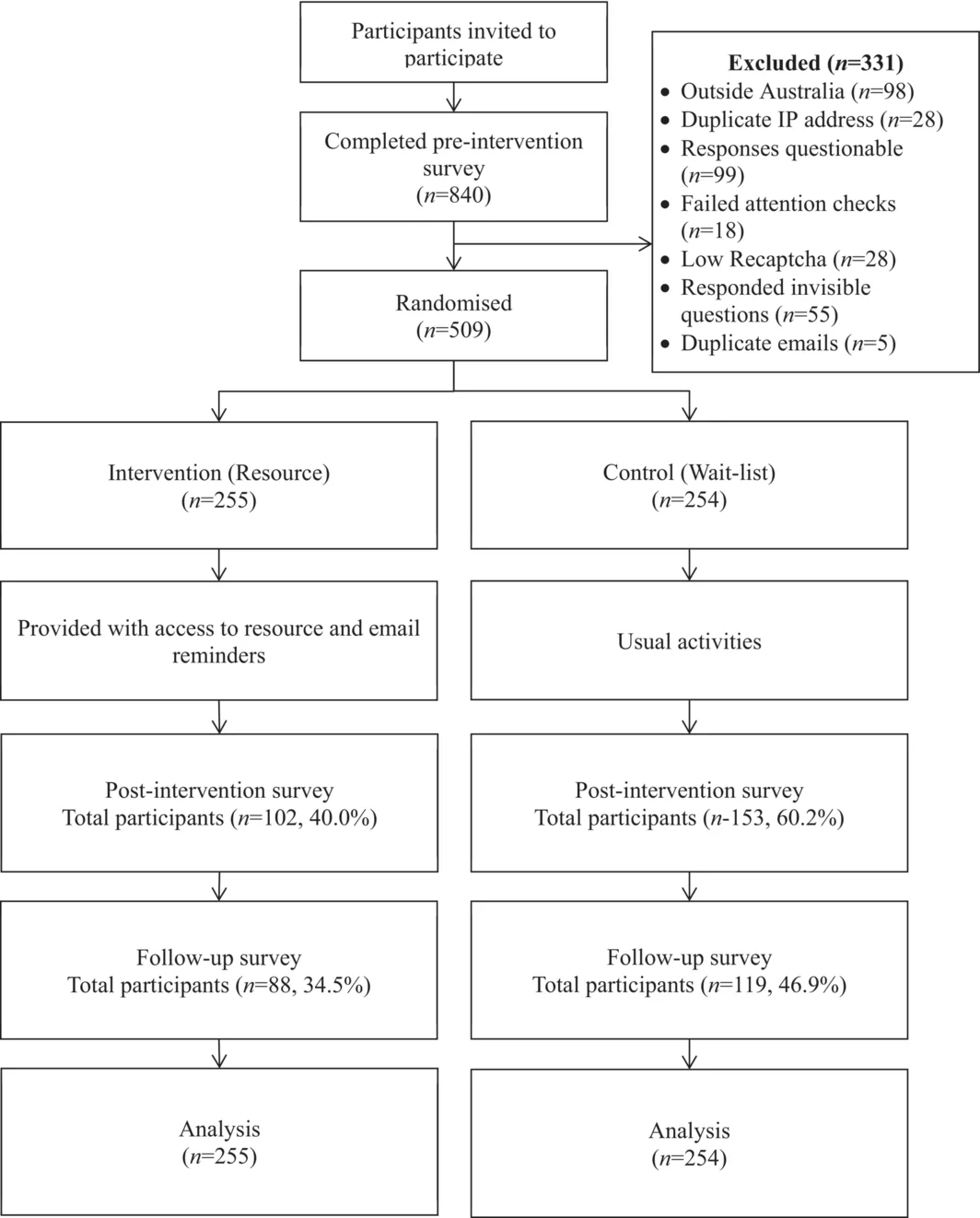
CONSORT flowchart

### Measures

#### Demographic variables

A range of demographic variables was assessed at pre‐intervention, including age, gender, language spoken at home, State/Territory of residence, location, employment status, level of education, relationship status, number of children, number of primary school‐ and/or secondary school‐aged children, previous qualifications or training in mental health, mental health history of the parent, mental health history and treatment seeking of the children and concern about their child's mental health in the past 3 months.

#### Self‐efficacy

Ten items adapted from the Parent Self‐Efficacy Scale (Czyz, Horwitz, Yeguez, Ewell Foster, & King, 2018) and Confidence in Helping Scale (Sebbens, Hassmén, Crisp, & Wensley, 2016) were used to assess self‐efficacy, with five items assessing mental health self‐efficacy and five assessing self‐harm and suicide self‐efficacy. Participants rated on an 11‐point scale ranging from 0 (Not at all confident) to 10 (Completely confident) their level of confidence in recognising signs and symptoms, initiating conversations, responding to disclosures, providing support to their child and seeking professional help. Higher scores indicated greater levels of self‐efficacy. The internal consistency of the mental health self‐efficacy (Cronbach's *α* = .87) and suicide self‐efficacy (*α* = .91) scales was good in the current study.

#### Perceived gatekeeper knowledge

A 15‐item bespoke measure based on the self‐evaluation of knowledge questions used in the Question, Persuade, Refer (QPR) gatekeeper training assessments (Tompkins & Witt, 2009) was used to assess perceived gatekeeper knowledge. Eleven items assessed perceived gatekeeper knowledge of mental health problems in children and adolescents, while the remaining four items assessed perceived gatekeeper knowledge associated with self‐harm and suicide. Items were rated on an 11‐point scale ranging from 0 (Very low) to 10 (Very high), with higher scores indicating greater levels of perceived knowledge. Both the mental health (*α* = .93) and suicide (*α* = .94) scales demonstrated good internal consistency in the current study.

#### Mental health and suicide literacy

Four items from the Anxiety Literacy Questionnaire (A‐Lit) (Gulliver et al., 2012) and four items from the Depression Literacy Questionnaire (D‐Lit) (Griffiths, Christensen, Jorm, Evans, & Groves, 2004) were used to assess participants' mental health literacy. Knowledge of suicide literacy was assessed with four items from the Literacy of Suicide Scale (LOSS) (Calear, Batterham, Trias, & Christensen, 2022). Items on all three scales were responded to on a 3‐point scale (True, False, Don't know), with correct responses allocated one point. Total scores on each scale ranged from 0 to 4, with higher scores indicating greater knowledge.

To assess specific knowledge of child and adolescent mental health, the 7‐item Parent Knowledge of Depression and Suicide Scale (PKDS) (Fox, Eisenberg, McMorris, Pettingell, & Borowsky, 2013) was also administered. Items on this measure are rated on a 4‐point scale ranging from ‘Strongly disagree’ to ‘Strongly agree’, with a fifth ‘Don't know’ response option. Correct responses are allocated a score of one, with total scores ranging from 0 to 7. Higher scores reflect greater knowledge.

#### Mental illness and suicide stigma

An adapted version of the 10‐item personal stigma sub‐scale of the Generalised Anxiety Stigma Scale (GASS) (Griffiths, Batterham, Barney, & Parsons, 2011) was used to assess mental illness stigma. In the adapted version of the scale, ‘anxiety disorder’ was replaced with ‘mental health problem’ in each item. Items are responded to on a 5‐point scale ranging from 0 (Strongly disagree) to 4 (Strongly agree), with total scale scores ranging from 0 to 40. The stigma (8 items) and glorification/normalisation (4 items) sub‐scales from the Stigma of Suicide Scale–Short Form (SOSS‐SF) (Batterham, Calear, & Christensen, 2013) were used to assess attitudes towards suicide. Participants rate their level of agreement with 1‐ to 2‐word descriptors of a person who dies by suicide on a 5‐point Likert scale ranging from 1 (Strongly disagree) to 5 (Strongly agree), with total scores ranging from 8 to 40 for the stigma sub‐scale and 4–20 for the glorification/normalisation sub‐scale. Higher scores on all scales are indicative of more negative attitudes. The internal consistency of the three scales was good (mental illness stigma *α* = .85; suicide stigma *α* = .90; suicide normalisation/glorification *α* = .86).

#### Help‐seeking attitudes, barriers and intentions

Help‐seeking attitudes were assessed using a modified version of the 10‐item Attitude Toward Seeking Psychological Professional Help Scale – Short form (ATSPPH‐SF) (Calear, Batterham, & Christensen, 2014; Fischer & Farina, 1995). The scale was modified to elicit a parent's attitude towards help‐seeking for their child. Each item on the scale was rated on a 4‐point Likert scale ranging from 0 (Disagree) to 3 (Agree), with total scale scores ranging from 0 to 30. Higher scores indicate more positive attitudes towards seeking professional help. Internal consistency of the ATSPPH‐SF was acceptable (*α* = .69).

To assess parent perceptions of modifiable barriers to help‐seeking, a list of 11 barriers was developed based on items from the Barriers to Access to Care Evaluation (BACE) (Clement et al., 2012), and previous research investigating help‐seeking barriers (Cairns, Potter, Nicholas, & Buhagiar, 2019; Czyz, Horwitz, Eisenberg, Kramer, & King, 2013). Each item is responded to on a 5‐point scale ranging from 1 (Not true for me) to 5 (True for me), with total scale scores ranging from 11 to 55. Higher scores indicate higher perceived barriers to help‐seeking. The internal consistency of the barriers to help‐seeking measure was good (*α* = .83).

The General Help‐Seeking Questionnaire (GHSQ) (Wilson, Deane, Ciarrochi, & Rickwood, 2005) was used to measure help‐seeking intentions for child personal or emotional problems from 12 different sources. Items were responded to on a 7‐point Likert scale ranging from 1 (Extremely unlikely) to 7 (Extremely likely). The factor structure of the GHSQ was examined using an exploratory principal components analysis, identifying three factors with loadings of ≥0.6. Two items (intimate partner and phone helpline) had low extraction values and were therefore excluded. The three factors identified were family and friends (child's grandparent, other relative/family member, friend), health professionals (general practitioner, paediatrician, psychologist, psychiatrist) and school staff (child's teacher, executive teacher, school counsellor). Higher total scale scores for each factor indicated greater help‐seeking intentions. Internal consistencies were acceptable for family and friends (*α* = .70), health professionals (*α* = .73) and school staff (*α* = .87).

#### Psychological distress

The 5‐item Distress Questionnaire‐5 (DQ5) (Batterham et al., 2016) was used to assess participants' general psychological distress in the past 30 days. Items were rated on a scale from 1 (Never) to 5 (Always), with total scale scores ranging from 5 to 25. Higher scores are reflective of greater psychological distress. The internal consistency of the DQ5 was good (*α* = .84).

#### Resource satisfaction, acceptability and engagement

Among intervention condition participants, the level of engagement with the resource was assessed using resource backend data (e.g. logins and completion). At post‐intervention, participants reported on the number of times they accessed the resource, time spent on the resource, whether they downloaded any information sheets and whether they shared the resource content with anyone. Open‐ended responses were reviewed to examine the different ways participants had used and shared materials from the resource. Satisfaction and acceptability of the resource were also assessed at post‐intervention using a satisfaction measure adapted from a previous study (March, Spence, & Donovan, 2009).

### Procedure

Upon recruitment, all potential participants were provided with a link to an online portal which hosted information about the trial, confirmed trial eligibility, collected digital consent and administered the pre‐intervention survey. To commence the pre‐intervention survey, participants were required to provide their email address to enable enrolment in the online resource and administration of post‐intervention and follow‐up surveys.

Once randomised, participants allocated to the intervention condition were provided with instructions and login details to access the resource via a web‐based content learning management system (CMS). Weekly email reminders were sent over 4 weeks to the intervention participants who had not accessed the resource. During the intervention phase of the trial, participants in the wait‐list control condition were instructed to continue with their usual activities.

After the four‐week intervention period, all participants were invited by email to complete the post‐intervention survey. Participants were invited to complete the final self‐report survey at 12‐weeks post‐intervention (with completion between January 2024 and January 2025). Up to two email reminders were sent to participants at each time point if surveys weren't completed. Surveys took participants approximately 20–30 min to complete. To assist with participant retention in the trial, all participants were offered a $15 e‐gift card for completing the post‐intervention and follow‐up surveys. Upon completion of the trial period, participants in the wait‐list control condition were provided access to the resource for 4 weeks.

### Statistical analysis

#### Attrition

Logistic regression analyses were conducted to identify significant predictors of attrition at post‐intervention and 12‐week follow‐up.

#### Intervention effects

All analyses of continuous measures were conducted on an intent‐to‐treat basis, including all participants randomised regardless of intervention completion or withdrawal from the study. Mixed‐model repeated‐measures (MMRM) ANOVA was used, with time as a within‐groups factor and condition as a between‐groups factor. This approach yields unbiased estimates of intervention effects under the assumption that data is missing completely at random or at random and allows the inclusion of participants with missing data without using biased techniques, such as last observation carried forward (Molenberghs & Verbeke, 2000). For any dichotomous outcomes, a comparable binary mixed modelling approach (Rabe‐Hesketh, Skrondal, & Gjessing, 2008) was used. Relationships between observations at different occasions were modelled using an unstructured covariance matrix. Robust standard error estimates were used (through the *GENLINMIXED* procedure) to account for the possibility of non‐normal residuals. Error degrees of freedom were calculated with Satterthwaite's method. The raw residuals of each model were examined with normal probability plots and were found to be within acceptable limits. The *p*‐value was not adjusted for multiple comparisons because adjustments were not pre‐specified in the protocol (Calear et al., 2024) based on the recommendation of balancing statistical significance with the magnitude of effects obtained and risk of Type II errors (Feise, 2002). Cohen's *d* effect sizes were calculated for all outcomes at each time point. Exploration of potential moderator effects was separately tested using three‐way interactions between intervention condition, time and moderator, with three potential moderators tested: age, gender and having a child with a mental health problem. All analyses were planned a priori and were conducted using SPSS Version 28.0 for Windows.

#### Satisfaction, acceptability and engagement

Descriptive statistics were used to summarise resource use, resource satisfaction and acceptability.

## Results

### Participant characteristics

A total of 509 parents and caregivers (96.9% female) participated in the trial. Table 1 presents participant characteristics by trial condition. Participant ages ranged from 24 to 66 years, with a mean age of 43.24 years (*SD* = 6.29). Participants were recruited from across Australia, with the highest proportion located in New South Wales (NSW; 27.2%) and Victoria (19.9%). At pre‐intervention, the majority (90.4%) of participants reported only speaking English at home and 58.8% reported living in a metropolitan location. Most (85.5%) participants were currently in paid work, 76.7% were married or in a de facto relationship and 75% had completed a tertiary qualification. Just under half of participants (47.2%) reported having two children and most (77.2%) reported having children of primary school age (5–12 years). Approximately half of the participants (49.9%) reported having not received any qualifications and/or training in mental health and 70.5% reported a personal history of mental health problems. More than two‐thirds reported that their child had experienced a mental health problem, with 84% receiving treatment. Close to three‐quarters (74.3%) of participants reported being concerned about their child's mental health in the past 3 months.

**Table 1:** Participant characteristics at baseline by trial condition

Variable	Intervention (*N* = 255)		Control (*N* = 254)	
	Number	%	Number	%
Gender				
Male	7	2.7	8	3.1
Female	248	97.3	245	96.5
Other identity	0	0	1	0.4
Language at home				
English only	230	90.2	229	90.5
Other language	25	9.8	24	9.5
State/Territory				
Australian Capital Territory	34	13.4	32	12.6
New South Wales	76	29.9	62	24.4
Northern Territory	3	1.2	1	0.4
Queensland	33	13	40	15.7
South Australia	21	8.3	16	6.3
Tasmania	9	3.5	6	2.4
Victoria	48	18.9	53	20.9
Western Australia	30	11.8	44	17.3
Location				
Metropolitan	148	58	150	59.5
Regional	85	33.3	83	32.9
Rural/remote	22	8.6	19	7.5
Currently in paid work	217	86.1	212	84.8
Education				
Some or all high school	10	3.9	11	4.3
Certificate/diploma	54	21.3	52	20.5
Bachelor's degree	87	34.3	89	35
Higher degree	103	40.6	102	40.2
Relationship status				
Single (never married)	11	4.4	13	5.2
Married or de facto	195	78	190	75.4
Divorced/separated	2	0.8	2	0.8
Widowed	42	16.8	47	18.7
Number of children				
One child	54	21.2	46	18.1
Two children	111	43.5	129	50.8
Three or more children	90	35.3	79	31.1
Primary school‐aged children	200	78.4	193	76
High school‐aged children	117	45.9	140	55.1
No MH qualifications/training	130	51.0	124	48.8
Participant history MH problems	184	72.2	175	68.9
Child history MH problems	172	67.5	172	67.7
Child received MH treatment^a^	150	87.2	139	80.8
Concern for child MH past 3 months	184	72.2	194	76.4
	***M***	***SD***	***M***	***SD***
Age	42.96	6.36	43.53	6.21
Psychological distress (DQ5)	12.06	3.71	12.02	3.61

MH, mental health.

^a^ Question only presented to participants reporting history of child MH problems.

### Attrition

A CONSORT flowchart of participant progression through the study is provided in Figure 1. Rates of attrition were higher than expected and significantly different across the intervention and wait‐list control conditions, with greater attrition in the intervention condition. At post‐intervention and follow‐up, attrition was only significantly associated with trial condition, with no differences based on gender, age, history of child mental health problems, or pre‐intervention levels of psychological distress, self‐efficacy, or perceived knowledge.

### Primary and secondary outcomes

The outcomes of the trial are summarised in Tables 2 and 3. Table 2 displays the overall model significance, as well as the condition‐by‐time interactions at post‐intervention and follow‐up from the MMRM analyses for each of the outcomes examined. Table 3 presents the estimated marginal means (and standard errors) for the intervention and wait‐list control conditions at post‐intervention and follow‐up from the MMRM analyses and the Cohen's *d* between‐group effect sizes calculated from raw means (see Table S1) at each time‐point. For the primary outcomes of mental health self‐efficacy and suicide self‐efficacy, significant intervention effects were observed at post‐intervention but not at the 12‐week follow‐up. In relation to the secondary outcomes, significant intervention effects were observed for perceived knowledge of mental health and suicide and help‐seeking barriers at both post‐intervention and follow‐up, while suicide literacy and suicide stigma only demonstrated significant positive effects at post‐intervention. No intervention effects were observed for mental health literacy, anxiety literacy, depression literacy, mental health stigma, suicide glorification/normalisation, or help‐seeking attitudes and intentions.

**Table 2:** Effect of condition and time on trial outcomes

Outcome	Model	Condition × post‐intervention			Condition × follow‐up		
		*B*	*t*	*p*	*B*	*t*	*p*
Self‐efficacy – Mental health	*F*(2, 241.3) = 2.07, *p* = .13	**1.67**	**2.03**	**0.04**	0.91	1.03	0.30
Self‐efficacy – Suicide	*F*(2, 258.1) = 3.14, *p* = .04	**2.54**	**2.44**	**0.01**	1.83	1.89	0.06
Perceived knowledge – Mental health	*F*(2, 254.0) = 8.77, *p* < .001	**7.26**	**4.14**	**<0.001**	**4.17**	**2.28**	**0.02**
Perceived knowledge – Suicide	*F*(2, 261.5) = 13.39, *p* < .001	**4.80**	**5.13**	**<0.001**	**3.46**	**3.76**	**<0.001**
Literacy – Anxiety	*F*(2, 252.9) = 0.77, *p* = .46	0.06	0.81	0.42	−0.03	−0.40	0.69
Literacy – Depression	*F*(2, 228.4) = 1.45, *p* = .24	0.09	1.70	0.09	0.05	0.83	0.41
Literacy – Depression & suicide	*F*(2, 263.8) = 2.61, *p* = .08	0.17	1.50	0.13	−0.10	−0.80	0.42
Literacy – Suicide	*F*(2, 262.2) = 3.13, *p* = .04	**0.31**	**2.49**	**0.01**	0.20	1.49	0.14
Stigma – Mental health	*F*(2, 266.6) = 1.71, *p* = .18	−0.69	−1.85	0.07	−0.19	−0.51	0.61
Stigma – Suicide	*F*(2, 247.2) = 4.78, *p* = .01	**−1.25**	**−2.41**	**0.02**	0.05	0.08	0.93
Stigma – Suicide glorification	*F*(2, 246.8) = 0.01, *p* = .99	0.04	0.12	0.91	−0.02	−0.06	0.95
Help‐seeking – Attitudes	*F*(2, 260.0) = 0.85, *p* = .43	0.46	1.29	0.20	0.18	0.43	0.67
Help‐seeking – Barriers	*F*(2, 258.3) = 3.55, *p* = .03	**−1.71**	**−2.43**	**0.02**	**−1.72**	**−2.23**	**0.03**
Help‐seeking – Intentions (family & friends)	*F*(2, 265.2) = 1.39, *p* = .25	0.58	1.66	0.10	0.33	0.83	0.41
Help‐seeking – Intentions (health professionals)	*F*(2, 270.7) = 0.07, *p* = .93	0.06	0.13	0.89	−0.13	−0.23	0.82
Help‐seeking – Intentions (school staff)	*F*(2, 270.8) = 0.38, *p* = .68	0.10	0.25	0.80	−0.28	−0.62	0.54

*B* = unstandardised estimate from MRMM analyses on each outcome that included all main effects and time × condition interactions. Bold values indicate significant effects (*p* < 0.05).

**Table 3:** Estimated marginal means (SE) based on mixed model repeated measures for each condition at each time point, with effect sizes

Outcome	Condition	Baseline	Post	3 months	Cohen's d effect size	
		*M* (SE)	*M* (SE)	*M* (SE)	Post	3 months
Self‐efficacy – Mental health	Intervention	35.81 (0.56)	38.66 (0.68)	39.08 (0.72)	0.33	0.24
	Control	34.08 (0.56)	35.26 (0.59)	36.44 (0.64)		
Self‐efficacy – Suicide	Intervention	31.88 (0.70)	36.48 (0.84)	37.53 (0.82)	0.40	0.35
	Control	29.37 (0.70)	31.43 (0.73)	33.19 (0.74)		
Perceived knowledge – Mental health	Intervention	73.08 (1.29)	86.12 (1.42)	86.37 (1.49)	0.51	0.35
	Control	69.45 (1.29)	75.24 (1.25)	78.57 (1.33)		
Perceived knowledge – Suicide	Intervention	21.73 (0.65)	28.40 (0.76)	28.99 (0.75)	0.54	0.42
	Control	20.36 (0.65)	22.24 (0.66)	24.17 (0.67)		
Literacy – Anxiety	Intervention	3.36 (0.04)	3.47 (0.06)	3.40 (0.06)	0.07	−0.03
	Control	3.36 (0.04)	3.41 (0.05)	3.43 (0.05)		
Literacy – Depression	Intervention	3.87 (0.03)	3.93 (0.04)	3.90 (0.04)	0.16	0.08
	Control	3.86 (0.03)	3.83 (0.04)	3.85 (0.04)		
Literacy – Depression & suicide	Intervention	6.00 (0.06)	6.31 (0.09)	6.17 (0.09)	0.21	0.00
	Control	5.90 (0.07)	6.04 (0.07)	6.17 (0.08)		
Literacy – Suicide	Intervention	2.71 (0.07)	3.18 (0.10)	3.16 (0.10)	0.27	0.18
	Control	2.64 (0.07)	2.79 (0.08)	2.89 (0.09)		
Stigma – Mental health	Intervention	3.34 (0.26)	2.99 (0.36)	3.60 (0.36)	0.13	0.14
	Control	3.92 (0.26)	4.27 (0.32)	4.37 (0.32)		
Stigma – Suicide	Intervention	12.17 (0.31)	11.35 (0.46)	12.04 (0.48)	0.18	0.01
	Control	12.17 (0.31)	12.61 (0.41)	12.00 (0.43)		
Stigma – Suicide glorification	Intervention	8.70 (0.21)	8.63 (0.30)	8.38 (0.33)	0.02	0.03
	Control	8.82 (0.21)	8.71 (0.26)	8.53 (0.29)		
Help‐seeking – Attitudes	Intervention	24.41 (0.22)	24.78 (0.29)	24.58 (0.34)	0.14	0.05
	Control	24.30 (0.22)	24.21 (0.25)	24.30 (0.30)		
Help‐seeking – Barriers	Intervention	21.16 (0.49)	18.77 (0.61)	19.10 (0.70)	0.19	0.16
	Control	21.16 (0.49)	20.48 (0.54)	20.81 (0.63)		
Help‐seeking – Intentions (family & friends)	Intervention	13.10 (0.26)	13.83 (0.30)	13.69 (0.33)	0.15	0.08
	Control	13.00 (0.26)	13.16 (0.26)	13.27 (0.29)		
Help‐seeking – Intentions (professionals)	Intervention	20.40 (0.29)	21.32 (0.37)	21.18 (0.42)	0.05	0.01
	Control	20.18 (0.29)	21.05 (0.31)	21.09 (0.37)		
Help‐seeking – Intentions (school staff)	Intervention	15.40 (0.26)	15.81 (0.33)	15.76 (0.35)	0.02	−0.05
	Control	15.42 (0.26)	15.72 (0.28)	16.05 (0.31)		

### Moderator analyses

Across all primary and secondary outcomes, only three moderation effects were significant. However, all effects were weak (*p* ≥ 0.035) and inconsistent across related measures of mental health literacy. These reflected greater increases from baseline to follow‐up in mental health literacy among parents who did not have a child with a mental health problem compared to those who did in the control group (*F* = 3.241, *df* = 2, 242.5, *p* = .041); greater decreases from post‐test to follow‐up in suicide literacy among men compared to women in the intervention group (*F* = 3.285, *df* = 2, 278.5, *p* = .039); and greater increases in the PKDS from baseline to follow‐up among men compared to women in the intervention group (*F* = 3.390, *df* = 2, 270.3, *p* = .035).

### User engagement, satisfaction and acceptability

Based on resource engagement data, 24.7% of participants completed the resource, 30.2% partially completed the resource and 45.1% did not start the resource. Of those participants who completed the post‐intervention survey, 92.2% had accessed the resource. The number of times participants accessed the resource ranged from 1 to 20 times, with most (84%) engaging with the resource over 1–4 sessions. The median amount of time spent on the resource was 56 min (*M* = 108.88, *SD* = 124.83).

Eighty‐two participants in the intervention condition provided feedback on the resource. Approximately three‐quarters (74.1%) of these participants reported that the resource met their needs, 91.4% would recommend it to a friend and 92.6% were satisfied with it. Additionally, 85.2% found the resource useful in teaching them skills to recognise mental health problems, 82.7% found it useful to talk to their children about mental health problems and 83.9% found it useful in teaching skills associated with seeking professional help. Just over half of participants (53.7%) reported downloading help‐sheets or summaries from the resource, with the vast majority doing so for future reference. At post‐intervention, 29.3% of participants reported having shared something they had learned from the resource with someone they knew, including partners, grandparents and friends. No adverse effects were reported in the trial.

## Discussion

The findings of the current study partially support the first hypothesis, with the intervention showing positive effects on parent and caregiver self‐efficacy to recognise, respond and access support for mental health problems and suicide risk in their child at post‐intervention, but these effects were not maintained at 12‐week follow‐up. There was also mixed support for the second hypothesis, with positive intervention effects observed at post‐intervention and follow‐up for perceived mental health and suicide knowledge and help‐seeking barriers, but for suicide stigma and literacy at post‐intervention only. No significant effects were observed for any other outcomes. Negligible evidence for moderation effects was found, suggesting that outcomes were largely consistent across age, gender and those with a child with versus without a mental health problem.

The positive effects observed in the current study broadly align with the literature, which has found gatekeeper programs to increase participant confidence and literacy and decrease stigma (McKay et al., 2022; Torok et al., 2019; Yonemoto et al., 2019). A novel finding in the current study was that the resource had positive effects on help‐seeking barriers, suggesting the resource may assist users in better navigating the help‐seeking process. Similar to previous studies with parents and caregivers (McKay et al., 2022), the effect of the resource on self‐efficacy was higher at post‐intervention than follow‐up. Based on previous research, longer–term self‐efficacy may be bolstered in future through the introduction of booster sessions to remind users of the key learnings of the resource (Yani, Chua, Wong, Pikkarainen, & Shorey, 2025). Instructional videos could also be used in the resource to solidify learning (Morgado et al., 2024) by demonstrating key skills, such as how to respond to disclosures of self‐harm or suicide. Providing participants with the opportunity to practice the skills learned through interaction with a chatbot may also be a novel way to increase self‐efficacy (Laun & Wolff, 2025). Innovative use of technology and AI to enhance learning and self‐efficacy should be explored in future training models. The effects for suicide and self‐harm also tended to be stronger than those obtained for mental health. This may reflect parents' lower awareness of suicide and self‐harm, compared to common mental disorders and the potentially higher levels of stigma associated with suicide and self‐harm. It may also reflect the high proportion of participants in the current trial with primary school‐aged children, for whom the prevalence of suicide and self‐harm is low and thus their awareness of these issues may be low too.

The lack of intervention effects on anxiety and depression literacy, mental health stigma and help‐seeking attitudes and intentions may be due to the presence of ceiling and floor effects on these variables at pre‐intervention, resulting in little room for improvement. The high levels of mental health literacy and low levels of stigma exhibited by participants may reflect the high level of education among the sample (75% tertiary educated) or their relatively high exposure to mental health problems, with 70% of participants reporting a personal history of mental health problems and over two thirds reporting that their child had mental health problems. Relatedly, 84% of participants who reported a child with a mental health problem had accessed treatment, suggesting an openness to help‐seeking.

These findings may also suggest a gap between knowledge and perceived ability to implement the knowledge, with more intensive interventions or greater time required to establish behaviour change. Future research could explore how interventions might better support this group by focusing on enhancing confidence, consolidating existing knowledge and building on strengths rather than solely providing information. Approaches such as personalised coaching, interactive or scenario‐based practice and opportunities for tailored feedback could help parents apply their knowledge in real‐life situations and feel more empowered in their role. Assessing the effect of such enhancements among parents with limited prior exposure to mental health issues would also provide insight into how interventions can balance the needs of both highly engaged and less experienced caregivers.

There are several strengths associated with the current study. This is one of the first controlled studies of a self‐directed gatekeeper intervention designed specifically for parents and caregivers and evaluated with parents of children and adolescents. The high proportion of participants with primary school‐aged children may reflect the perceived relevance and urgency of learning about mental health and suicide prevention for younger children, as parents of this age group may be particularly motivated to develop skills early in their child's development. Parents may also see primary school years as a critical period for recognising early signs of mental health difficulties and establishing supportive communication patterns, making them more likely to engage with preventive resources. Potential implications of this research therefore may include the promotion and delivery of the resource in schools to upskill parents and caregivers in how to best support their child's mental health. Clinicians may also consider providing the resource to families following a new diagnosis or as part of safety planning for a young person identified at‐risk of suicide or self‐harm. The sample obtained in the current study was also geographically diverse, with broad representation across Australia and in metropolitan and rural areas.

Alongside these strengths are some study limitations. Just over 50% of participants in the intervention condition started the resource and only 25% completed the resource. Top reasons for not accessing the resource included forgetting to do the resource, not prioritising the completion of the resource and not having enough time to engage with the resource. Relatedly, there was also a high level of drop‐out for post‐intervention and follow‐up survey completion in the intervention condition, with most of these participants not accessing the intervention. This suggests that people who did not engage with the resource were reluctant to complete follow‐up surveys. The mixed level of engagement with the intervention may have diluted potential intervention effects, while the high attrition rate may have resulted in insufficient power to detect some intervention effects. There would be value in exploring the effects of adherence in future studies. More importantly, though, is the need to find ways to better engage people in preventive health interventions and assist them in prioritising this activity. This is particularly imperative for parents, who are often time poor but may require the knowledge and skills to adequately support their children. Other, more accessible, mobile models, such as using podcasts to deliver the same information, may be a worthy approach to trial, enabling parents to multitask in the face of time constraints. Another potential limitation of the study is the representativeness of the sample in terms of gender, cultural diversity, exposure to mental health disorders and previous mental health qualifications and training. It was very difficult to recruit male participants to the trial, and thus, the efficacy of the resource in this population is less clear. People with a lived experience of mental health problems and/or those with mental health qualifications or previous mental health training were disproportionally attracted to the current study. There would be value in tailoring strategies in future studies to identify and engage parents and caregivers who are less familiar with mental health or are yet to access mental health services, to better understand the impact of the resource on outcomes where ceiling or floor effects were observed. A lack of cultural diversity was evident in the sample, with the majority of participants identifying as English‐speaking. Future research should prioritise the inclusion of participants from diverse cultural backgrounds to ensure the resource is accessible, culturally appropriate and effective across different communities.

## Conclusion

Overall, the results of the current trial are positive, with the resource demonstrating effects on self‐efficacy, perceived knowledge, barriers to help‐seeking and suicide stigma and literacy at post‐intervention, with only some effects maintained at follow‐up. The resource had stronger effects on suicide outcomes, likely reflecting the community's lower awareness and exposure to suicide compared to common mental disorders. Future research should explore strategies and intervention approaches to improve engagement, particularly among parents and caregivers with limited previous exposure to mental health and suicide risk.

## Ethical considerations

Participants provided informed consent to participate in the study. This study received ethics approval in 2023 from the Australian National University's Human Research Ethics Committee (Protocol 2023/195).

**Key points**

**What's known?**

- Parents and caregivers play key roles in recognising and responding to mental distress in their children.
- Gatekeeper training interventions improve confidence and knowledge to respond to at‐risk individuals, yet few have been developed for or evaluated with parents.

**What's new?**

- A newly developed gatekeeper resource for parents was effective at improving mental health self‐efficacy (*p* = .04) and suicide self‐efficacy (*p* = .01) at post‐intervention.
- Significant intervention effects were also found for perceived knowledge (*p* < .001), barriers to help‐seeking (*p* = .02), suicide stigma (*p* = .02) and literacy (*p* = .01) at post‐intervention, with some effects maintained at 12‐week follow‐up.

**What's relevant?**

- Gatekeeper resources targeted to parents and caregivers can be effective in improving their confidence and knowledge to support their child's mental health.

## Supporting information


**Table S1.** Observed Means (SD) for each condition at each timepoint.

## Data Availability

The data that support the findings of this study are available on request from the corresponding author. The data are not publicly available due to privacy or ethical restrictions.
