# Supplementary material for: Randomised controlled trial of a web‐based mental health, self‐harm and suicide gatekeeper resource for parents and caregivers
Source: J Child Psychol Psychiatry. 2026 Jun 12;67(11):1844–54. doi: 10.1111/jcpp.70189 (PMC13647100; doi:10.1111/jcpp.70189)
Supplement: Supplementary file 1 [file JCPP-67-1844-s001.docx]

**Randomised controlled trial of a web-based mental health, self-harm and suicide gatekeeper resource for parents and caregivers**

**Supporting Information**

**Table S1. Observed Means (SD) for each condition at each timepoint**

| **Outcome** | **Condition** | **Baseline (*n* = 509)** | **Post (*n* = 255)** | **3 months (*n* = 207)** |
| --- | --- | --- | --- | --- |
|  |  | **M (SD)** | **M (SD)** | **M (SD)** |
| Self-efficacy - Mental health | Intervention | 35.82 (8.52) | 38.25 (7.47) | 39.28 (7.68) |
|  | Control | 34.08 (9.18) | 36.31 (8.36) | 37.09 (8.70) |
| Self-efficacy - Suicide | Intervention | 31.87 (11.15) | 36.56 (8.74) | 38.02 (8.28) |
|  | Control | 29.37 (11.18) | 32.49 (10.36) | 34.02 (10.53) |
| Perceived knowledge – Mental health | Intervention | 73.08 (19.55) | 85.46 (16.30) | 86.70 (15.37) |
|  | Control | 69.45 (21.64) | 77.53 (17.13) | 80.77 (18.70) |
| Perceived knowledge – Suicide | Intervention | 21.70 (10.30) | 28.43 (7.93) | 29.33 (7.80) |
|  | Control | 20.36 (10.43) | 22.99 (9.57) | 25.18 (9.44) |
| Literacy – Anxiety | Intervention | 3.36 (0.58) | 3.47 (0.62) | 3.39 (0.61) |
|  | Control | 3.36 (0.61) | 3.39 (0.60) | 3.43 (0.66) |
| Literacy – Depression | Intervention | 3.87 (0.40) | 3.92 (0.39) | 3.88 (0.47) |
|  | Control | 3.86 (0.50) | 3.82 (0.54) | 3.87 (0.44) |
| Literacy – Depression & Suicide | Intervention | 6.00 (1.03) | 6.26 (0.88) | 6.17 (0.99) |
|  | Control | 5.90 (1.02) | 6.09 (0.96) | 6.20 (0.84) |
| Literacy – Suicide | Intervention | 2.71 (1.08) | 3.18 (1.01) | 3.11 (0.96) |
|  | Control | 2.64 (1.06) | 2.83 (1.12) | 2.87 (1.14) |
| Stigma – Mental health | Intervention | 3.34 (3.77) | 3.27 (4.09) | 4.00 (4.38) |
|  | Control | 3.92 (4.48) | 4.37 (5.29) | 4.58 (5.09) |
| Stigma – Suicide | Intervention | 12.16 (4.82) | 11.58 (5.56) | 12.19 (5.46) |
|  | Control | 12.17 (5.10) | 12.46 (5.87) | 12.24 (6.09) |
| Stigma – Suicide glorification | Intervention | 8.70 (3.45) | 8.43 (3.53) | 8.39 (3.64) |
|  | Control | 8.82 (3.27) | 8.78 (3.53) | 8.66 (3.63) |
| Help-seeking – Attitudes | Intervention | 24.41 (3.54) | 24.35 (3.45) | 23.86 (4.06) |
|  | Control | 24.30 (3.58) | 24.31 (3.43) | 24.35 (3.75) |
| Help-seeking – Barriers | Intervention | 21.16 (7.46) | 19.64 (7.63) | 19.94 (7.41) |
|  | Control | 21.16 (8.08) | 20.03 (7.57) | 20.59 (9.16) |
| Help-seeking – Intentions (Family & friends) | Intervention | 13.10 (4.14) | 13.48 (3.70) | 13.41 (4.05) |
|  | Control | 13.00 (4.02) | 13.14 (3.61) | 13.16 (3.35) |
| Help-seeking – Intentions (Professionals) | Intervention | 20.40 (4.61) | 21.15 (4.31) | 20.91 (4.64) |
|  | Control | 20.20 (4.56) | 21.00 (4.04) | 21.02 (4.42) |
| Help-seeking – Intentions (School staff) | Intervention | 15.40 (4.16) | 15.66 (4.04) | 15.72 (4.19) |
|  | Control | 15.42 (4.11) | 15.73 (3.69) | 16.18 (3.61) |
